# Nanobubble-mediated sonodynamic therapy enhances cuproptosis in the treatment of hepatocellular carcinoma†

**DOI:** 10.1039/d5na00280j

**Published:** 2025-06-12

**Authors:** Jiabao Ouyang, Nan Li, Chunbo Liu, Yu Zhang

**Affiliations:** a Department of Ultrasound, The First Affiliated Hospital of Harbin Medical University No. 199, Xidazhi Road, Nangang District City Harbin Heilongjiang Province 150001 China oyjb317939191@163.com +86 15645084968

## Abstract

*Background and objective*: Hepatocellular carcinoma (HCC) is one of the primary causes of cancer-associated death worldwide, which has few viable therapeutic options at later stages. Cuproptosis, a newly discovered kind of programmed cell death, gives a new therapeutic target for HCC. Nanobubble (NB)-mediated sonodynamic therapy (SDT) has been a prospective method for augmenting the efficacy and delivery of therapeutic agents. This study investigates the potential of NB-mediated SDT to enhance cuproptosis in HCC treatment, aiming to evaluate the efficacy of NB-mediated SDT in enhancing cuproptosis in HCC cells and to clarify the potential mechanisms. *Methods*: NBs were engineered to encapsulate the sonosensitizer protoporphyrin IX (PPIX) and the cuproptosis inducer elesclomol-Cu^2+^ (ES-Cu), forming PPIX@ES-Cu NBs, a targeted delivery system activated by ultrasound. HCC cell lines (Hepa1-6) were treated with these PPIX@ES-Cu NBs followed by low-intensity ultrasound (LIUS) exposure. Mitochondrial function and cell viability were evaluated through CCK assays and fluorescence microscopy. qPCR was implemented for examining the expression of cuproptosis-associated genes. *Results*: The NB-mediated SDT significantly enhanced the delivery of copper ions and sonosensitizers to HCC cells. Upon ultrasound activation, there was a substantial increase in ROS production and intracellular copper accumulation. This led to mitochondrial dysfunction, characterized by decreased mitochondrial membrane potential and disrupted tricarboxylic acid (TCA) cycle enzyme activities. Treated cells exhibited increased expression of cuproptosis markers, including upregulation of lipoylated mitochondrial proteins and proteotoxic stress indicators. Cell viability assays demonstrated a considerable decrease in HCC cell survival compared to controls. *Conclusion*: NB-mediated SDT effectively enhances cuproptosis in HCC cells by facilitating targeted delivery and activation of therapeutic agents. The synergistic effect of ROS generation and copper accumulation induces cuproptosis, offering a novel and potent therapeutic strategy for HCC treatment. These results lay the groundwork for more *in vivo* research and possible clinical use of this combo treatment.

## Abstract 2

PPIX@ES-Cu NBs-mediated ultrasound-controlled enhanced cuproptosis for hepatocellular carcinoma.

## Graphical abstract

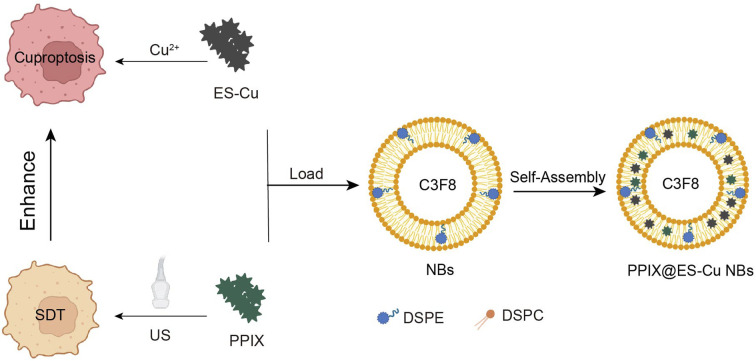

## Background

HCC is the most frequent primary liver cancer, and among the major causes of cancer-associated death around the world.^1^ Alcoholic liver disease, non-alcoholic fatty liver disease, and chronic hepatitis B and C infections are among the potential liver conditions that are frequently linked to HCC. The overall prognosis for individuals with HCC is still poor, despite advancements in treatment techniques, such as liver transplantation, surgical resection, and systemic therapies, including tyrosine kinase inhibitors and immune checkpoint inhibitors. Late-stage diagnosis, high rates of tumor recurrence, and resistance to conventional treatments underscore the need for innovative therapeutic strategies to improve patient survival.^2,3^

SDT is a novel non-invasive cancer therapy modality that exploits the synergistic interaction between ultrasound and sonosensitizers.^4–6^ Under LIUS, sonosensitizers are activated to generate ROS, which lead to oxidative stress and hence tumor cell death.^7^ SDT offers significant advantages over traditional therapies, such as its deep tissue penetration, precise localization of therapeutic effects, and minimal invasiveness.^8–10^ However, its clinical application is limited by challenges including inefficient delivery and activation of sonosensitizers, as well as insufficient tumor targeting.^11–13^

Recent advances in cancer biology have identified novel mechanisms of cell death that can be exploited for cancer treatment.^14^ One such mechanism is cuproptosis, a distinct programmed form of cell death, differentiated from necrosis, ferroptosis, and apoptosis.^15–17^ The accumulation of intracellular copper ions causes cuproptosis, which in turn causes cell death *via* directly binding to mitochondrial enzymes, interfering with the TCA cycle, and causing proteotoxic stress.^18–21^ HCC, a metabolically active tumor type reliant on mitochondrial function, is particularly vulnerable to cuproptosis, rendering it a prospective target for therapy intervention.^22–24^

To overcome the SDT limitations and augment the efficacy of cuproptosis therapy, NB technology has emerged as a versatile platform.^25,26^ NBs are gas-filled particles stabilized by lipid, protein, or polymer shells, designed to serve as both ultrasound contrast agents and drug delivery vehicles.^27,28^ When exposed to ultrasound, NBs undergo cavitation, releasing their therapeutic payload and inducing transient increases in vascular permeability, thus strengthening the delivery of copper ions and sonic sonosensitizers to the tumor microenvironment.^29,30^ Furthermore, NBs improve the localized activation of sonodynamic agents, amplifying ROS production and optimizing tumor cell death.^26^ By combining SDT with cuproptosis-inducing agents, a synergistic effect can be achieved, leveraging both oxidative stress and copper-mediated proteotoxicity to maximize tumor eradication.

Recent advances in cancer therapy have focused on enhancing treatment efficacy while minimizing side effects, and sonodynamic therapy (SDT) has emerged as a promising non-invasive modality that combines ultrasound with sonosensitizers to generate reactive oxygen species (ROS) and induce tumor cell death. However, the clinical application of SDT is still limited due to challenges such as inefficient delivery of sonosensitizers and inadequate tumor-specific activation. Copper-mediated therapies have shown promise for inducing cell death through copper accumulation, disrupting mitochondrial function, and generating oxidative stress. Yet, the combination of SDT with copper-based agents, particularly in the context of a novel programmed cell death mechanism known as cuproptosis, remains largely unexplored in hepatocellular carcinoma (HCC) treatment.

In this study, we present a novel therapeutic strategy by combining nanobubble (NB)-mediated SDT with cuproptosis-inducing agents (PPIX@ES-Cu), aiming to overcome the limitations of traditional SDT and copper-mediated therapies. The combination of these two therapies enhances the delivery of copper ions and sonosensitizers to HCC cells, while ultrasound activation amplifies ROS generation, inducing mitochondrial dysfunction and proteotoxic stress. By triggering cuproptosis, which is a distinct form of cell death, we explore a new approach to selectively target and eliminate HCC cells. This strategy offers an alternative to conventional cancer therapies, particularly for tumors like HCC that rely heavily on mitochondrial function.

## Materials and methods

### Preparation of PPIX@ES-Cu NBs

**Fig. 1:**
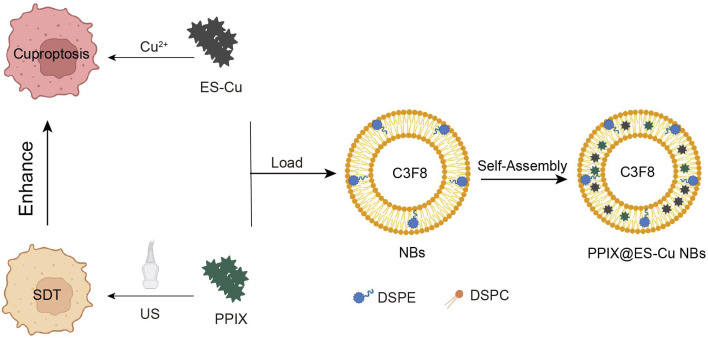
PPIX@ES-Cu NB-mediated ultrasound-controlled enhanced cuproptosis for hepatocellular carcinoma. This figure illustrates the mechanism by which PPIX@ES-Cu NBs, activated by ultrasound, enhance cuproptosis in Hepa1-6 cells. The synergistic effects of sonodynamic therapy (SDT) and cuproptosis induction are depicted, with an emphasis on targeted delivery and activation of the therapeutic agents.

PPIX@ES-Cu NBs were produced through a modified thin-film hydration approach^31^ (Fig. 1). A lipid mixture consisting of cholesterol (molar ratio 65 : 30 : 5), DSPE-PEG-2000 (1,2-distearoyl-*sn-glycero*-3-phosphoethanolamine-*N* [methoxy (polyethylene glycol)-2000]), and DPPC (1,2-dipalmitoyl-*sn-glycero*-3-phosphocholine) was dissolved in chloroform, and a thin film was developed by evaporation under decreased pressure. The films were hydrated in PBS (pH 7.4) *via* incorporating a sonosensitizer (PPIX) and a solution comprising a copper ion donor (CuCl_2_). The dried lipid film was resuspended in PBS to form a lipid suspension. The suspension was extruded 20 times *via* a 200 nm film using a micro-extruder (Avanti Polar Lipids, Alabaster, AL). The solution extruded was moved to a sealed vial after the syringe was emptied and injected with C3F8. The mixture was stirred utilizing a dental stirrer (YJT Medical Apparatus and Instruments, Shanghai, China) for 60 s, resuspended in PBS (2 ml) and stored at 4 °C.

### Characterization of PPIX@ES-Cu NBs

A TEM was used for observing the morphology of NBs. Dynamic light scattering (DLS) was applied to identify the NB zeta potential and size distribution. Stability studies were performed by storing NBs at 4 °C and analyzing their size over 48 hours. The absorption spectra of ES-Cu and PPIX@ES-Cu NBs were obtained using a UV-vis spectrometer (SHIMADZU, Japan).

### Ultrasound-triggered release of PPIX and ES-Cu

To quantify and visualize the ultrasound-triggered release of PPIX and ES-Cu from PPIX@ES-Cu NBs, fluorescence spectroscopy and UV-vis spectroscopy were used. PPIX@ES-Cu NBs at a concentration of XX μM were subjected to ultrasound exposure at 1 MHz, 1.0 W cm^−2^ for various durations (1, 3, 5, 10, and 15 minutes). After ultrasound treatment, the supernatant was collected for analysis. PPIX release was quantified using fluorescence spectroscopy, where fluorescence intensity was measured at 485 nm excitation and 520 nm emission wavelengths. ES-Cu release was quantified using UV-vis spectroscopy at 250 nm. A calibration curve was constructed for both PPIX and ES-Cu to convert fluorescence and absorption data into concentrations.

### Encapsulation efficiency of PPIX and ES-Cu in PPIX@ES-Cu NBs

The encapsulation efficiency of PPIX and ES-Cu in PPIX@ES-Cu NBs was determined by measuring the amount of each compound encapsulated within the nanobubbles and comparing it to the total amount used in the preparation. PPIX@ES-Cu NBs were prepared by loading PPIX and ES-Cu into the nanobubbles. To measure the encapsulation efficiency of PPIX, free PPIX was separated from the NBs by centrifugation, and the concentration of free PPIX in the supernatant was measured using fluorescence spectroscopy at an excitation wavelength of 485 nm and an emission wavelength of 520 nm. The encapsulated PPIX was calculated by subtracting the free PPIX concentration from the total amount of PPIX used in the preparation. For ES-Cu, the release was quantified by measuring the ES concentration in the supernatant using UV-vis spectroscopy at a wavelength of 250 nm.

## Cell culture

The mouse hepatocellular carcinoma cell lines (Hepa1-6) were obtained from Procell. The cells were cultivated in DMEM with 1% penicillin-streptomycin and 10% fetal bovine serum (FBS) at 5% CO_2_ humidity and 37 °C.

### *In vitro* CCK assay

Hepa1-6 cells were seeded in 6-well plates and incubated overnight to achieve 70–80% confluence. Then, the cells were treated with various concentrations of PPIX@ES-Cu NBs. Following 4 h of incubation, the cells were subjected to ultrasound (duration: 5 min, intensity: 1.0 W cm^−2^, frequency: 1 MHz). The viability of the cells was examined *via* the CCK assay following treatment.

### Dead and live cell staining

Post-treatment cell viability was evaluated using the Live/Dead Cell Dual-Fluorescence Staining Kit (Calcein-AM/Propidium Iodide, PI) to distinguish viable cells (stained green by calcein-AM) from non-viable cells (stained red by PI).^32^ Hepa1-6 cells were cultivated overnight at 37 °C with 5% CO_2_ in DMEM with 1% penicillin-streptomycin and 10% FBS after being seeded into 6-well plates at 1 × 10^5^ cells per well. The cells were separated into five groups, including SDT, NC, SDT + ES-Cu + ATTM, SDT + ES-Cu, and ES-Cu. Prior to ultrasonic treatment, the cells in the treated groups were cultivated for four hours. Following treatment, the cells were rinsed in PBS and stained utilizing a solution made from serum-free DMEM containing 4 μM PI and 2 μM Calcein-AM. To enable dye uptake, staining was conducted at a temperature of 37 °C for 20 minutes under darkness. After incubation, the cells were rinsed in PBS once more to get rid of extra dye. They were then examined right away using an inverted fluorescence microscope equipped with TRITC and FITC filters to visualize dead cells (red fluorescence from PI) and living cells (green fluorescence from Calcein-AM).

### Flow cytometry assay

In accordance with the manufacturer's protocol, apoptosis was examined using an Annexin V-FITC/PI Apoptosis Detection Kit.^32^ Hepa1-6 cells were cultivated in DMEM with 1% penicillin-streptomycin and 10% FBS in a 37 °C incubator with 5% CO_2_ for the whole night after being inoculated in 6-well plates at 2 × 10^5^ cells per well. The cells were categorized into five groups: NC, SDT, ES-Cu, SDT + ES-Cu, and SDT + ES-Cu + ATTM. Cells in the treated groups were incubated for 4 hours prior to ultrasound exposure. After treatment, the cells were collected by trypsinization and centrifuged at 1000 × *g* for 5 minutes. The cell pellets were washed twice with cold PBS and resuspended in 500 μL of 1× binding buffer provided in the kit. To each sample, 5 μL of PI together with 5 μL of Annexin V-FITC was incorporated. After carefully mixing the samples, they were incubated at ambient temperature for 15 minutes under darkness. After incubation, the labeled cells were examined immediately *via* a flow cytometer (BD FACSCanto II).

Measurement of fluorescent signals was carried out in PI (red) and FITC (green) channels to differentiate between live (Annexin V−/PI−), late apoptotic (Annexin V+/PI+), early apoptotic (Annexin V+/PI−) and necrotic (Annexin V−/PI+) cells. FlowJo software was used to analyze data gathered from a minimum of 10 000 events per sample. The fraction of both early and late apoptotic cells was applied to compute apoptotic indices. Each experiment was implemented in triplicate, and the results were represented as mean ± SD. Tukey's post-hoc test and one-way ANOVA were used for the statistical analysis, and a *p*-value below 0.05 was deemed statistically significant.

### ROS assay

The effect of PPIX@ES-Cu NBs on the production of ROS (reactive oxygen species) in the Hepa1-6 cells was investigated using a ROS assay kit (Beyotime Institute of Biotechnology). In 12-well plates, Hepa1-6 cells were sown in triplicate and allowed to develop to 40–50% confluency. NC, SDT, ES-Cu, SDT + ES-Cu, and SDT + ES-Cu + ATTM were then added to the cell plate, which was then incubated for six hours at 37 °C with 5% CO_2_ in the air. The cells were treated and then incubated for 20 minutes at 37 °C with a 10 μM DCFH-DA probe. The fluorescence signal intensities representing ROS levels were then quickly inspected *via* a fluorescent microscope (Axiovert 200, Zeiss, Germany).

### Assessment of mitochondrial membrane potential (MMP)

A JC-1 detection kit (Beyotime, C2005) was used for evaluating MMP.^33^ After culturing 5 × 10^5^ cells per well of Hepa1-6 cells into 6-well plates, the cells were treated as per the experimental groups (NC, SDT, ES-Cu, SDT + ES-Cu, and SDT + ES-Cu + ATTM) for 24 hours. After treatment, the cells were stained *via* JC-1 for 20 minutes, and later they were cleaned twice with buffer solution. The cells were then stained with JC-1 and were characterized *via* fluorescence microscopy (Axiovert 200, Zeiss, Germany).

## Intracellular GSH assay

The GSH level in Hepa1-6 cells was examined *via* a GSH Assay Kit (Beyotime, Shanghai, China). In 6-well plates (3 × 10^5^ per well), the cells were treated with NC, SDT, ES-Cu, SDT + ES-Cu, and SDT + ES-Cu + ATTM respectively. The intracellular level of GSH was then assessed using the GSH Assay.

### Assay for MDA

As per the manufacturer's instructions (Beyotime Biotechnology, Shanghai, China), the content of MDA was determined using a lipid peroxidation MDA assay kit (#S0131S). Cell lysates collected 24 hours after different treatments (NC, SDT, ES-Cu, SDT + ES-Cu, and SDT + ES-Cu + ATTM) were centrifuged at 10 000–12000*g* for 10 minutes at 4 °C to obtain the supernatant. Each standard (0.1 mL) or sample was mixed with 200 μL MDA solution, heated for 15 minutes at 100 °C, and later centrifuged for 10 minutes at 1000*g* to extract the supernatant. The supernatant (200 μL) was measured colorimetrically at OD = 532 nm using a Tecan Infinite M200, Switzerland.

### qRT-PCR with RNA extraction of cuproptosis

To evaluate the role of cuproptosis, cells were treated with PPIX@ES-Cu NBs, and total RNA was extracted, and an ABI 7500 Fast Real-Time PCR system (Applied Biosystems, USA) was used for qRT-PCR for identifying the expression levels of cuproptosis-associated proteins, encompassing LIAS and FDX1.

### Metabolic profiling and TCA cycle enzyme activity

To assess the impact of PPIX@ES-Cu NBs on the TCA cycle, the activity of key TCA cycle enzymes was measured following treatment and ultrasound exposure. Malate dehydrogenase (MDH) activity was measured according to the manufacturer's protocol (Elabscience). Briefly, after treatment, the cells were harvested and lysed, and enzyme activity was quantified by measuring the change in absorbance at 450 nm, which corresponds to the conversion of malate to oxaloacetate.

## Statistical analysis

All of the results are represented as mean ± SD. Group differences were evaluated using one-way ANOVA. *P* values of 0.05, 0.01, and 0.001 were utilized for determining significance.

## Results

### Characterization of PPIX@ES-Cu NBs

**Fig. 2:**
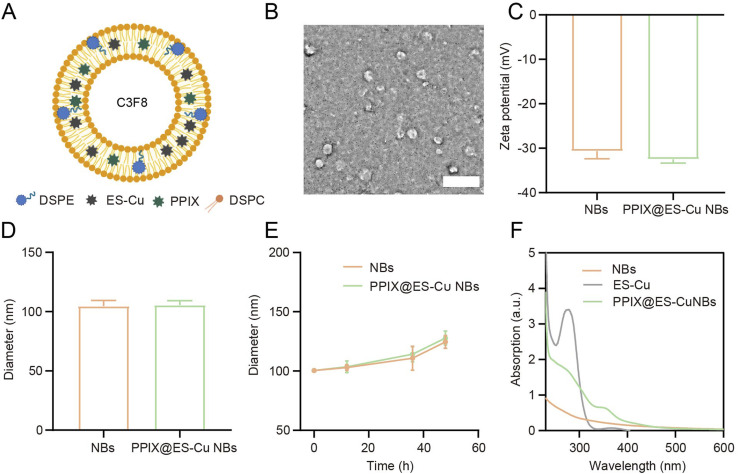
Synthesis and characterization of PPIX@ES-Cu NBs. (A) Schematic representation of the synthesis process, showing the intermolecular interactions between ES-Cu and PPIX in the formation of PPIX@ES-Cu NBs. (B) Transmission electron microscopy (TEM) image showing the spherical morphology of PPIX@ES-Cu NBs with an average diameter of approximately 100 nm (scale bar = 400 nm). (C) Dynamic light scattering (DLS) analysis revealing an average particle size of 105.6 ± 3.73 nm for PPIX@ES-Cu NBs. (D) Zeta potential measurement showing a value of −32.53 ± 0.81 mv, indicating the stability of PPIX@ES-Cu NBs. (E) Dimensional stability of PPIX@ES-Cu NBs over 2 days. (F) UV-vis spectra of PPIX@ES-Cu NBs, showing characteristic absorption peaks for both PPIX and ES-Cu.

The experimental design is summarized in the schematic workflow diagram (Fig. S1†), which outlines the key stages of the study. A step by step description is provided in the Results section. Under TEM, the PPIX@ES-Cu NBs revealed a consistent spherical morphology (Fig. 2B). Dynamic light scattering (DLS) measurements revealed an average particle size of 105.6 ± 3.73 nm and a narrow size distribution, indicating good homogeneity (Fig. 2C). The zeta potential was measured as −32.53 ± 0.81 mV, demonstrating the stability of the NBs (Fig. 2D). Stability studies confirmed that the NBs maintained their size and structural integrity over a 48-hour period when stored at 4 °C (Fig. 2E). The UV-vis spectrometry analysis confirmed the successful encapsulation of PPIX and ES-Cu, with characteristic absorption peaks corresponding to both compounds (Fig. 2F).

### Encapsulation efficiency of PPIX and ES-Cu in PPIX@ES-Cu NBs

The encapsulation efficiency of PPIX and ES-Cu in the PPIX@ES-Cu NBs was determined by measuring the amount of free PPIX and ES-Cu in the supernatant after centrifugation. For PPIX, the fluorescence intensity of the supernatant was measured, and the encapsulation efficiency was calculated by subtracting the free PPIX from the total amount used. The PPIX encapsulation efficiency was found to be approximately 85%. Similarly, the ES-Cu concentration in the supernatant was measured using UV-vis spectroscopy, and the encapsulation efficiency of ES-Cu was found to be around 80%. These results indicate that significant portions of both PPIX and ES-Cu were successfully encapsulated within the nanobubbles, ensuring effective loading for therapeutic applications.

### Ultrasound-triggered release of PPIX and ES-Cu

The ultrasound-triggered release of PPIX and ES-Cu from PPIX@ES-Cu NBs was successfully quantified using fluorescence spectroscopy and UV-vis spectroscopy. As shown in Fig. S2,† the release of PPIX increased in a time-dependent manner with prolonged ultrasound exposure. Similarly, the release of ES-Cu was measured by UV-vis spectroscopy, with ES concentration increasing with longer ultrasound exposure times. At 1 minute of ultrasound exposure, a modest increase in ES-Cu concentration (8 μM) was observed, which progressively increased up to 80 μM after 15 minutes of ultrasound exposure. The data confirmed a clear, time-dependent release of copper ions from PPIX@ES-Cu NBs, which was significantly enhanced with ultrasound exposure.

### *In vitro* cell viability

**Fig. 3:**
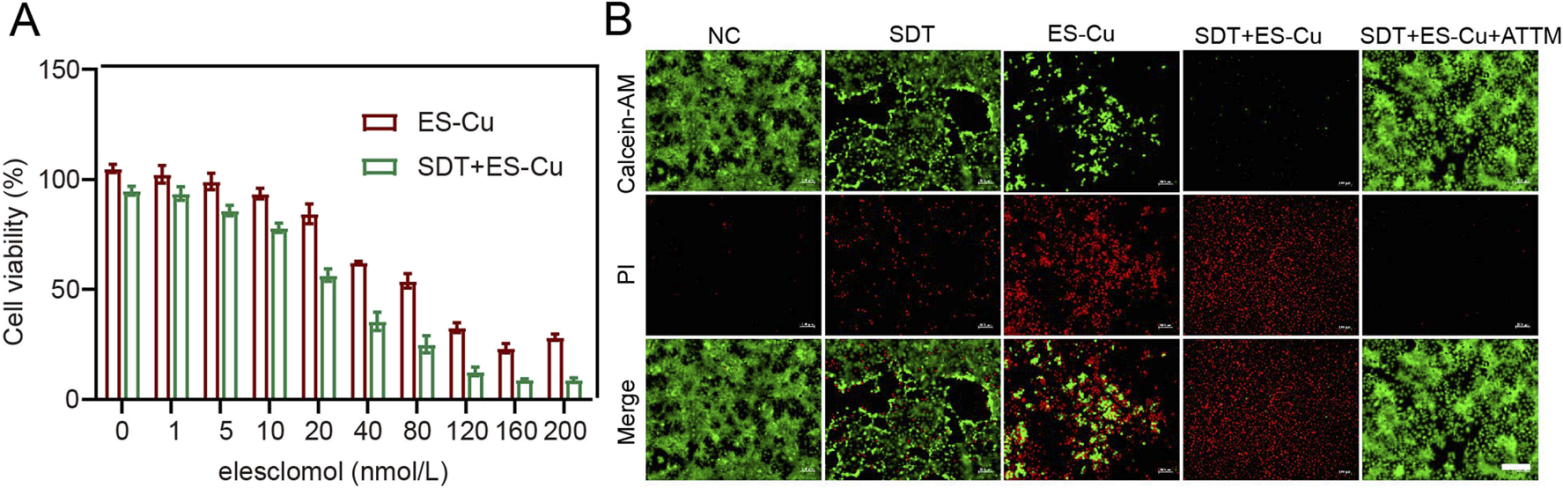
Cytotoxicity analysis of synergistic therapy. (A) Viability of Hepa1-6 cells after incubation with different treatments at different concentrations for 24 h. Data are given as mean ± s.d., *n* = 3 independent experiments. Statistical significance was determined using one-way ANOVA. (B) Representative fluorescence images (scale bar = 200 μm) of Hepa1-6 cells from various treatment groups after live/dead cell staining, with propidium iodide (red) marking dead cells and calcein AM (green) marking live cells.

CCK assay revealed a dose-dependent decrease in the viability of Hepa1-6 cells upon treatment with PPIX@ES-Cu NBs and ultrasound (SDT). The combination of SDT and ES-Cu exhibited significantly lower cell viability compared to the NC group, highlighting the synergistic cytotoxic effects (IC50 = 27.1 μM, Fig. 3A). Live/dead cell staining further confirmed these findings, with a marked increase in red fluorescence (dead cells) in the SDT + ES-Cu group in contrast to the NC, SDT, and ES-Cu groups (Fig. 3B). Addition of ATTM further reversed the cytotoxic effects, demonstrating that cuproptosis is enhanced by SDT forces.

### *In vitro* cell apoptosis

**Fig. 4:**
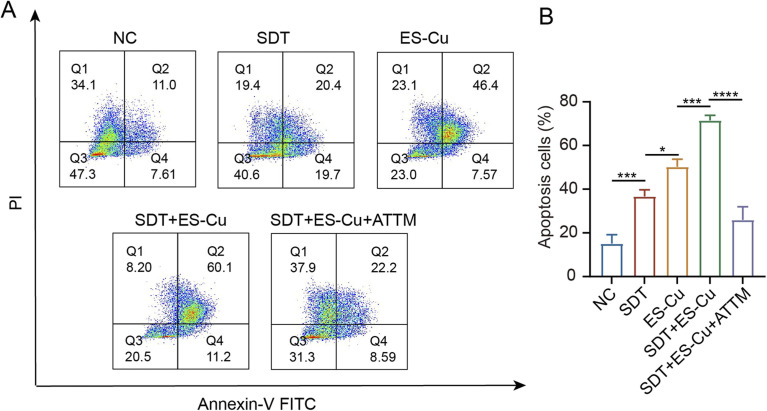
Apoptosis analysis of synergistic therapy. (A) Representative flow cytometry images showing the apoptosis status of Hepa1-6 cells from different treatment groups (SDT, ES-Cu, SDT + ES-Cu). (B) Apoptosis frequency of Hepa1-6 cells in various treatment groups, analyzed by flow cytometry. The data are expressed as mean ± s.d., *n* = 3 independent experiments. Statistical significance was evaluated by one-way ANOVA, with *p* < 0.05 indicating statistical significance.

Flow cytometry analysis of apoptosis revealed a significant rise in early and late apoptotic cells in the SDT + ES-Cu group compared to the NC group. The SDT + ES-Cu group had the highest apoptosis cells (sum of late and early apoptotic cells), indicating enhanced apoptosis induced by the combination therapy (Fig. 4A and B).

### Levels of ROS

Studies have shown that cuproptosis can effectively generate reactive oxygen species (ROS) in tumor cells, which plays a crucial role in inducing cell death by disrupting mitochondrial function and causing proteotoxic stress.^34^ The dichloro-dihydro-fluorescein diacetate (DCFH-DA) probe is widely used to assess ROS generation due to its ability to penetrate cell membranes and, upon oxidation by ROS, transform into dichloro-fluorescein (DCF), which exhibits strong green fluorescence. This fluorescent signal can be used to quantify and visualize ROS production within cells. In our study, Hepa1-6 cells treated with SDT + ES-Cu exhibited the highest levels of green fluorescence, confirming a substantial generation of ROS within the cells upon ultrasound-triggered activation of PPIX@ES-Cu NBs. The strong fluorescence intensity observed under fluorescence microscopy indicates that the combined effect of SDT and copper treatment greatly enhances ROS production, which contributes to the induction of cuproptosis. In contrast, cells treated with SDT + ES-Cu + ATTM, where copper availability was chelated by the copper-binding agent ATTM, showed a marked reduction in green fluorescence, indicating a significant decrease in ROS generation (Fig. S3†). This reduction supports the hypothesis that copper is essential for the ROS production induced by cuproptosis, and its inhibition through ATTM suppresses the ROS-mediated cell death pathway.

### Levels of MMP

**Fig. 5:**
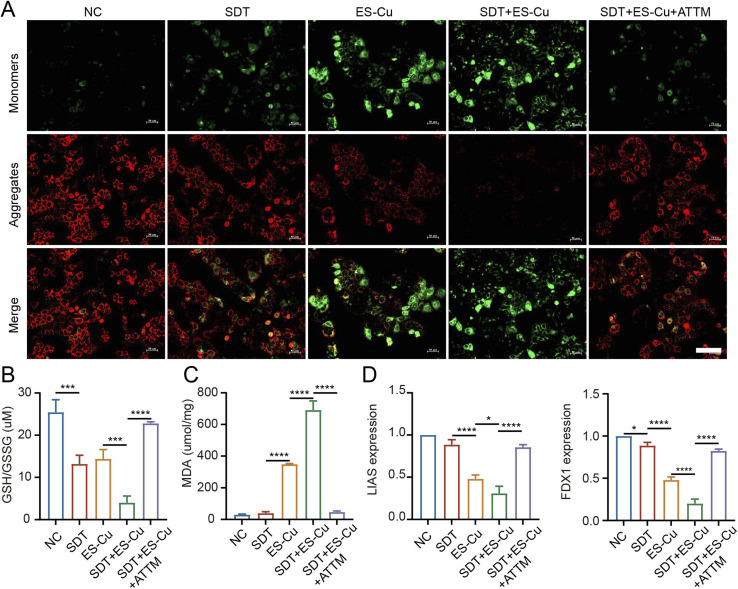
Functional analysis of PPIX@ES-Su NBs and MMP in cuproptosis. (A) Fluorescence microscopy images showing the aggregate (red channel) and JC-1 monomer (green channel) in the mitochondria of Hepa1-6 cells after treatment with different conditions (scale bar = 100 μm). Mitochondrial membrane potential (mmp) disruption is evidenced by the increase in green fluorescence. (B) MDA analysis showing the levels of lipid peroxidation in Hepa1-6 cells treated with various conditions. The data are shown as mean ± s.d. For three independent experiments. Statistical significance was determined by one-way ANOVA, with ****p < 0.0001 and ***p < 0.001. (C) GSH assay results showing the intracellular glutathione (GSH) levels in Hepa1-6 cells after different treatments, expressed as mean ± s.d. for three separate experiments. Statistical significance was determined using one-way ANOVA, with ****p < 0.0001 and ***p < 0.001. (D) PCR analysis of cuproptosis-related genes (FDX1, LIAS) in Hepa1-6 cells under various treatments. Data are presented as mean ± sd, *n* = 3 independent experiments. Statistical significance was determined using one-way ANOVA, with *p < 0.05 and ****p < 0.0001.

As displayed by JC-1 staining, the MMP (Δ*Ψ*_m_) was severely disrupted in cells with SDT + ES-Cu treatment, as evidenced by increased green fluorescence (monomeric JC-1) and decreased red fluorescence (aggregated JC-1). Upon incorporation of ATTM, this effect was reversed (Fig. 5A). The NB-mediated SDT significantly enhanced the delivery of copper ions and sonosensitizers to HCC cells, with ultrasound activation leading to a substantial increase in ROS production and intracellular copper accumulation. These changes were directly linked to mitochondrial dysfunction. We observed a marked decrease in mitochondrial membrane potential (MMP), as evidenced by JC-1 staining, with an increase in the green fluorescence of monomeric JC-1 and a decrease in red fluorescence from aggregated JC-1, indicating mitochondrial depolarization.

### Levels of GSH and MDA

The enhanced ROS generation upon ultrasound activation of PPIX@ES-Cu NBs further exacerbated oxidative stress, as indicated by a significant depletion of intracellular glutathione (GSH) levels, along with a marked increase in malondialdehyde (MDA) levels, a byproduct of lipid peroxidation. These results suggest that oxidative stress plays a pivotal role in the observed cytotoxicity. The SDT + ES-Cu group exhibited a substantial decrease in intracellular GSH levels *versus* the NC group, and this depletion was exacerbated in the SDT + ES-Cu + ATTM group, indicating enhanced oxidative stress (Fig. 5B). Comparable outcomes were seen for the level of intracellular MDA, the most common byproduct of lipid peroxidation and a direct indicator of the degree of lipid peroxidation (Fig. 5C).

### Gene expression of cuproptosis-related proteins

These mitochondrial changes were correlated with the upregulation of cuproptosis markers, including increased lipoylation of mitochondrial proteins. The increased lipoylation is consistent with the activation of cuproptosis, a copper-mediated form of programmed cell death. In addition, qRT-PCR analysis demonstrated remarkable deregulation of cupping-associated genes, including FDX1 and LIAS, in the SDT + ES-Cu group compared to NC, SDT, and ES-Cu alone, suggesting a mechanistic link between the treatment and the activation of cuproptosis pathways (Fig. 5D). This indicates that the combination of SDT and copper accumulation activates cuproptosis in HCC cells.

### Disruption of TCA Cycle enzyme activity

We assessed the activity of malate dehydrogenase (MDH), a key enzyme in the TCA cycle, to evaluate the impact of PPIX@ES-Cu NBs on mitochondrial metabolism. Treatment with PPIX@ES-Cu NBs followed by ultrasound exposure led to a significant decrease in MDH activity compared to the control group (Fig. S4†). This reduction in enzyme activity indicates that ultrasound-triggered PPIX@ES-Cu NBs disrupt the normal function of the TCA cycle in Hepa1-6 cells, consistent with our hypothesis of mitochondrial dysfunction as a mechanism of cuproptosis.

Thus, the combination of enhanced ROS generation and copper-induced proteotoxicity resulted in mitochondrial dysfunction and apoptosis, providing strong evidence that NB-mediated SDT effectively enhances cuproptosis in HCC cells.

## Discussion

Our study demonstrates the synergistic potential of combining NB-mediated SDT with cuproptosis-inducing agents (PPIX@ES-Cu) for the treatment of hepatocellular carcinoma (HCC). While SDT and copper-mediated therapies have been separately explored in cancer treatment, their combination, particularly through the induction of cuproptosis, represents a significant advancement. Previous studies on SDT have primarily focused on enhancing sonosensitizer delivery and ROS generation through ultrasound activation, but the clinical translation of SDT has been hindered by inefficient targeting and activation. Similarly, copper-based therapies have shown promise for inducing cell death by disrupting mitochondrial function, yet their potential remains underutilized in combination with SDT.

The novelty of our approach lies in the synergistic effects of combining SDT with cuproptosis. By using NBs to deliver both copper ions and sonosensitizers directly to the tumor site, and activating them through ultrasound, we achieve a targeted and efficient therapy. The ultrasound-mediated cavitation of NBs not only enhances drug delivery but also amplifies ROS production, which further sensitizes tumor cells to the cuproptosis-inducing agents. Unlike conventional forms of programmed cell death such as apoptosis or ferroptosis, cuproptosis occurs through mitochondrial dysfunction and proteotoxic stress, which makes it a promising therapeutic pathway for HCC. This novel mechanism of action provides an alternative to traditional cancer therapies, especially for liver cancers like HCC, which are highly dependent on mitochondrial function.

In summary, our study highlights the advantages of combining NB-mediated SDT with cuproptosis-inducing agents in HCC treatment. This innovative approach overcomes the limitations of previous therapies by enhancing targeted delivery, amplifying ROS generation, and exploiting a novel form of cell death. These findings set the stage for further research into the clinical application of this strategy, offering a potential therapeutic option for HCC patients and other cancers that rely on mitochondrial function.

While the results of this study highlight the potential of PPIX@ES-Cu NBs for enhancing sonodynamic therapy in HCC, several challenges must be addressed for their successful clinical translation. One major hurdle is the optimization of large-scale synthesis and production of PPIX@ES-Cu NBs. The current preparation methods, although effective for research purposes, require refinement to ensure the reproducibility, quality, and scalability necessary for clinical use. In addition, while NBs have shown excellent stability *in vitro*, their *in vivo* biodistribution and long-term stability remain critical factors for clinical translation. Efficient tumor targeting and minimizing non-specific uptake by the mononuclear phagocyte system (MPS) will be essential to avoid premature clearance and reduce off-target effects. Furthermore, the optimization of ultrasound parameters for clinical applications presents another challenge. The frequencies and intensities used in our study, although effective in preclinical models, may need to be adjusted according to individual patient anatomy and tumor characteristics to ensure maximum therapeutic efficacy without causing tissue damage. Lastly, the safety of PPIX@ES-Cu NBs, particularly regarding the long-term effects of copper accumulation and ROS generation in healthy tissues, requires comprehensive preclinical toxicity studies. These studies are crucial to assess any potential immunogenic responses and to evaluate the safety profile of this novel treatment. Addressing these challenges through further preclinical and clinical investigations will be vital to advancing the therapeutic potential of PPIX@ES-Cu NBs for the treatment of HCC.

## Conclusion

This work demonstrates the potential of NB-mediated sonodynamic therapy to enhance cuproptosis in HCC treatment. The combination of SDT and cuproptosis achieves synergistic effects by simultaneously inducing oxidative stress through reactive oxygen species and disrupting mitochondrial function *via* copper-mediated proteotoxicity. *In vitro* experiments showed significant reductions in cell viability, along with enhanced mitochondrial dysfunction and apoptosis.

The findings highlight the therapeutic advantages of leveraging NBs for targeted drug delivery and ultrasound-mediated activation, offering a novel and effective approach to HCC management. This novel approach has a lot of potential for clinical use and may be expanded to treat more solid cancers. The focus of future research should be on the optimization of NB design, the exploration of combinatorial regimens, and the conduct of clinical trials to validate these preclinical findings.

## Author contributions

J. O. performed conceptualization. J. O. and N. L. performed the investigation. C. L. wrote the original draft. J. O. and C. L. validated the software. C. L. and Y. Z. performed writing reviews and editing. J. O. performed supervision and project administration. The article was reviewed by all authors.

## Conflicts of interest

The authors have not disclosed any conflicts of interest.

## Supplementary Material

NA-OLF-D5NA00280J-s001

## Data Availability

The data supporting the plots within this paper and other study findings are available from the corresponding authors upon reasonable request.
