# Supplementary material for: Nanobubble-mediated sonodynamic therapy enhances cuproptosis in the treatment of hepatocellular carcinoma
Source: Nanoscale Adv. 2025 Jun 12;7(15):4651–9. doi: 10.1039/d5na00280j (PMC12184190; doi:10.1039/d5na00280j)
Supplement: NA-007-D5NA00280J-s001 [file NA-007-D5NA00280J-s001.pdf]

Supplementary Information (SI) for Nanoscale Advances.
This journal is © The Royal Society of Chemistry 2025

Supplementary Materials for

Nanobubbles-mediated sonodynamic therapy enhances cuproptosis in the treatment of hepatocellular carcinoma

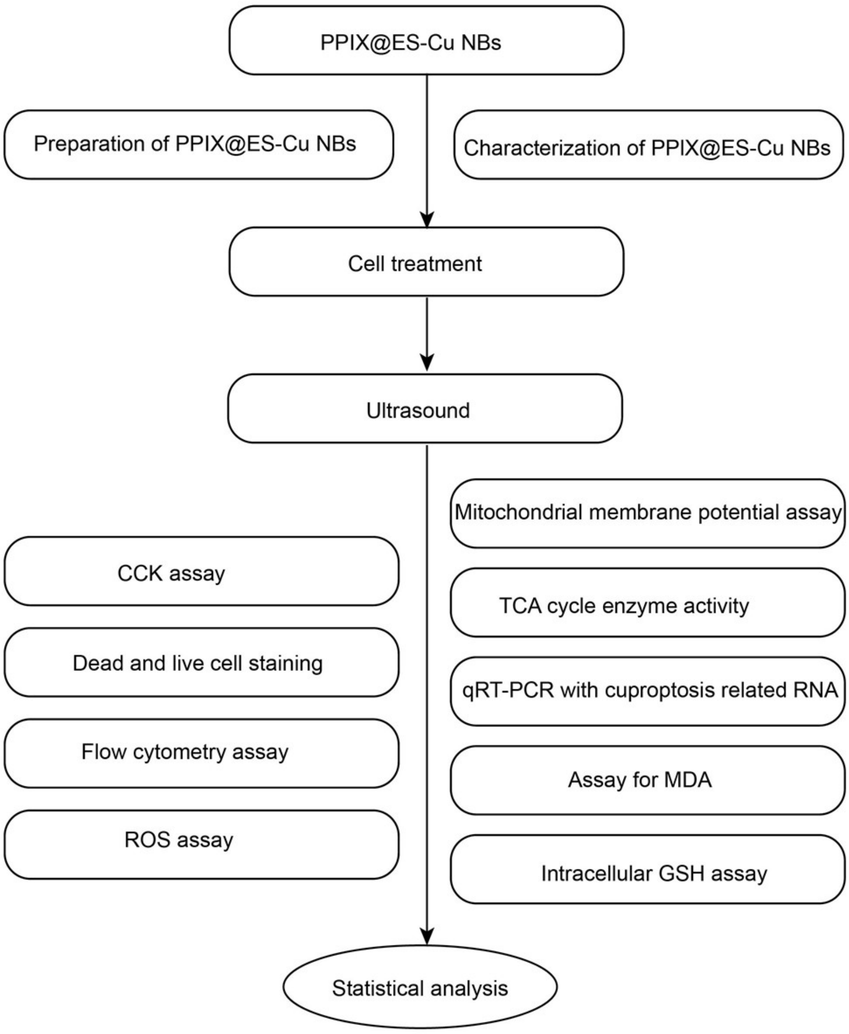

Figure S1. Schematic workflow summarizing the experimental design. The diagram outlines the key stages of the study to assess the effects of PPIX@ES-Cu NBs and ultrasound treatment on Hepa1-6 cells. The workflow includes steps such as cell treatment, ultrasound application, assessment of ROS generation using the DCFH-DA probe, cell viability assays, apoptosis analysis, metabolic profiling, and TCA cycle enzyme activity measurement. Final data analysis was performed using statistical methods to evaluate the effects of the treatments on cell function and death pathways.

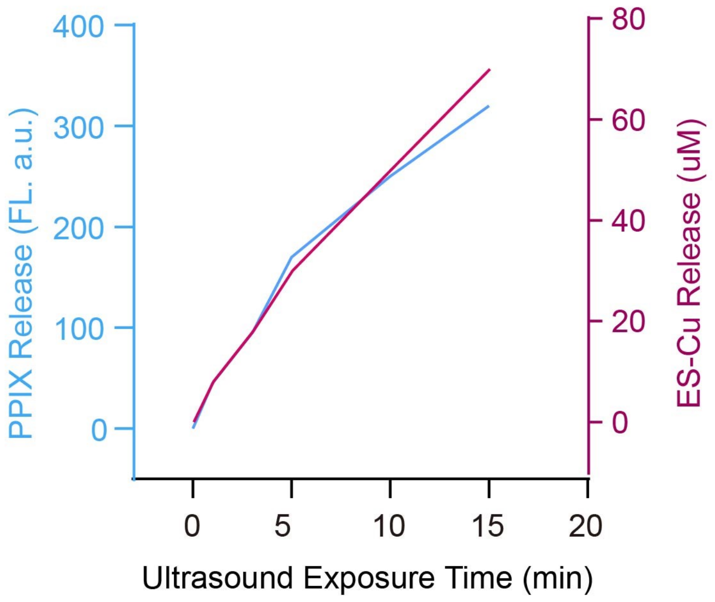

Figure S2. Ultrasound-triggered release of PPIX and ES-Cu. PPIX release was quantified using fluorescence spectroscopy, showing a time-dependent increase in fluorescence intensity as the ultrasound exposure time increased. ES-Cu release was quantified by UV-Vis spectroscopy, with copper ion concentration (μM) increasing progressively with ultrasound activation.

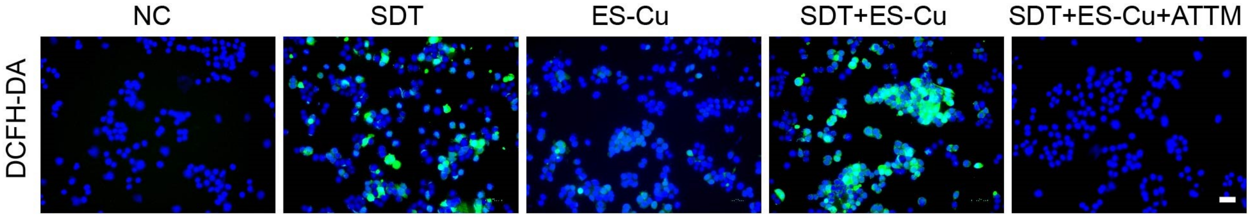

Figure S3. ROS generation measured by DCFH-DA staining in Hepa1-6 cells following ultrasound exposure.

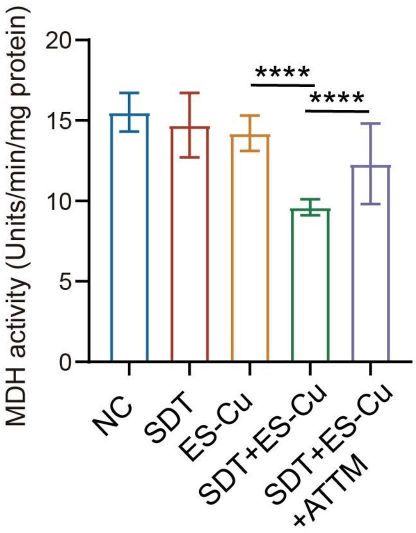

Figure S4. Impact of PPIX@ES-Cu NBs on TCA Cycle Enzyme Activity.
